# Expression of microsomal triglyceride transfer protein in lipoprotein-synthesizing tissues of the developing chicken embryo^☆^

**DOI:** 10.1016/j.biochi.2013.12.020

**Published:** 2014-06

**Authors:** Christine Eresheim, Julia Plieschnig, N. Erwin Ivessa, Wolfgang J. Schneider, Marcela Hermann

**Affiliations:** Max F. Perutz Laboratories, Department of Medical Biochemistry, Medical University of Vienna, Dr. Bohr-Gasse 9/2, 1030 Vienna, Austria

**Keywords:** Microsomal triglyceride transfer protein, Apolipoprotein B, Yolk sac, Lipoprotein, chicken, Development, Embryo

## Abstract

In contrast to mammals, in the chicken major sites of lipoprotein synthesis and secretion are not only the liver and intestine, but also the kidney and the embryonic yolk sac. Two key components in the assembly of triglyceride-rich lipoproteins are the microsomal triglyceride transfer protein (MTP) and apolipoprotein B (apoB). We have analyzed the expression of MTP in the embryonic liver, small intestine, and kidney, and have studied the expression of MTP in, and the secretion of apoB from, the developing yolk sac (YS). Transcript and protein levels of MTP increase during embryogenesis in YS, liver, kidney, and small intestine, and decrease in YS, embryonic liver, and kidney after hatching. In small intestine, the MTP mRNA level rises sharply during the last trimester of embryo development (after day 15), while MTP protein is detectable only after hatching (day 21). In the YS of 15- and 20-day old embryos, apoB secretion was detected by pulse-chase metabolic radiolabeling experiments and subsequent immunoprecipitation. Taken together, our data reveal the importance of coordinated production of MTP and apoB in chicken tissues capable of secreting triglyceride-rich lipoproteins even before hatching.

## Highlights

• MTP is expressed in liver, small intestine, and kidney of chicken embryos.
• MTP is expressed in the chicken yolk sac.
• ApoB is secreted from the chicken yolk sac.
• Embryonic tissues contribute to the lipoprotein pool of the developing chick.

## 1 Introduction

The major structural protein of triglyceride-rich lipoproteins secreted by the liver and intestine is apolipoprotein B (apoB). The microsomal triglyceride transfer protein (MTP) plays a major role in the synthesis and secretion of apoB-containing lipoproteins [1]. MTP is located in the lumen of the endoplasmic reticulum (ER) and is involved in the initial loading of nascent lipoproteins with neutral lipids, particularly triglycerides and cholesterol esters. Alternatively, or additionally, MTP may participate in the intricate process of apoB translocation across the ER membrane [2]. In humans, MTP has been shown to be involved in the biogenesis of lipoproteins containing apolipoprotein B100 in the liver and apolipoprotein B48 in the small intestine [3–5]; for review see Ref. [6].

Studies of human embryos showed expression of apoB RNA in the fetal liver, fetal intestine, and in the yolk sac (YS), but not in other embryonic tissues [7–9]. MTP and apoB are also expressed in the human placenta, suggesting that this organ can also synthesize and secrete apoB-containing lipoproteins [10]. During mouse embryonic development, MTP is expressed in the YS as well as in liver and small intestine [11,12], but not in cardiac and renal tissues. In the laying hen, it has been shown that MTP is expressed in tissues involved in lipid metabolism, i.e., the liver, small intestine, and the kidney [13]. Chicken liver synthesizes and secretes apoB100-containing lipoproteins, but apoB48 production is totally absent in any organ of chickens. Interestingly, chicken small intestine secretes so-called portomicrons, which are apoB100-containing lipoproteins [14,15].

Genetic deficiency of MTP in humans results in the autosomal recessive disease, abetalipoproteinemia [16,17]. The pathophysiology of this inherited condition may be attributed, at least in part, to the impaired absorption of the fat-soluble vitamins A and E [18,19]. Gene defects leading to structurally altered apoB secreted from the liver or intestinal mucosa results in hypobetalipoproteinemia [20–24], which also is associated with fat-soluble vitamin deficiencies in humans. In liver and intestine of heterozygous MTP knockout mice, the MTP mRNA and protein levels were reduced by 50%, and a marked reduction in total plasma cholesterol levels was observed. Studies of MTP knockout mouse embryos showed an impaired capacity of the YS to export lipids to the developing embryo and demonstrated an accumulation of cytosolic lipid droplets in the endodermal cells of the yolk sac, followed by lethal developmental abnormalities [25].

The chicken YS plays a crucial role in embryo growth, as it supplies the growing embryo with nutrients taken up from the yolk. Acquisition of this function is a process which is coordinated with that of vascularization of the YS [26]. MTP has been identified in chicken YS [26,27], but the expression of MTP in fetal tissues has not been studied in detail yet. We now show that MTP is expressed not only in the YS of the developing chicken embryo, but also in fetal liver, intestine, and kidney. Secretion of newly synthesized apoB in ex-vivo cultured YS tissue, which also synthesizes MTP, is compatible with the notion that this tissue exports triglyceride-rich lipoproteins to the embryonic circulation.

## 2 Material and methods

### 2.1 Animals

Sexually mature Derco brown (TETRA-SL) laying hens and roosters were purchased from Diglas Co. (Feuersbrunn, Austria) and maintained on open floor space with free access to water and feed (standard diet, Ssniff, Germany) under a daily light period of 16 h. For fertilized eggs, hens and roosters were housed together in flocks in our animal facility. Freshly fertilized eggs available in house were incubated at 37.5 °C and 60–70% humidity to maintain normal embryonic development. Pre-hatch eggs were removed from the incubator after 5, 7, 9, 10, 11, 13, 15, 17, 20 days; 1-day-old and 3-day-old chicks were also utilized. For tissue and organ retrieval, the chicks were euthanized by decapitation. The shell of eggs incubated for up to 20 days was cut open to expose the embryo for tissue preparation (liver, kidney, and small intestine); the YS membranes were excised, washed with ice-cold phosphate-buffered saline (PBS), and protease inhibitors (Complete, Roche, Manheim, Germany) were added to PBS buffer prior to the preparation of Triton X-100 protein extracts. For tissue and organ retrieval, the laying hens were euthanized by decapitation. All animal procedures were approved by the “Animal Care and Use Committee” of the Medical University of Vienna.

### 2.2 Preparation of antibodies

Polyclonal antibodies directed against a chicken MTP C-terminal fragment [13], and against chicken apoB100 [28] were raised by a standard procedure in adult female New Zeeland White rabbits in our laboratory. Antisera were tested by Western blotting.

### 2.3 Preparation of yolk sac layers and tissue culture

Chicken eggs were incubated to embryonic day (E) 9. YS tissue from E9 embryos was washed in PBS. Separation of layers was performed in PBS using watchmakers' forceps. Starting from the mesodermal layer containing blood vessels, the endodermal epithelial cell (EEC) layer and the mesoderm were peeled apart. The separated layers were immediately used for protein isolation.

For ex-vivo culture, yolk sacs were isolated from 15 or 20 days old embryos, and small pieces of 0.5–0.7 cm^2^ were cut off and incubated in DMEM d-Valine medium supplemented with 10% fetal calf serum, 2% horse serum, 100 units penicillin/ml, 0.1 mg/ml streptomycin, 1% fungizone and 2 mM l-glutamine, which was changed every day. The tissue fragments were incubated two days to remove the adherent yolk before use in experiments.

### 2.4 Metabolic labeling and immunoprecipitation

Cultured YS tissue specimens were washed in PBS and incubated in starvation medium (RPMI 1640 without l-glutamine, l-methionine, l-cysteine and l-cystine) for 1 h at 37 °C and 5% CO_2_. Thereafter, the medium was removed and the YS fragments were metabolically labeled in starvation medium with Met-label ([^35^S]methionine and [^35^S]cysteine) for 1 h. After the pulse phase, the labeling medium was removed and the YS tissue-pieces were incubated in medium as described above, supplemented with 200 mM l-glutamine, 400 mM l-methionine, 400 mM l-cysteine and l-cystine. After the indicated chase times, the supernatant was processed as follows. For immunoprecipitation, 500 μl of the supernatant were incubated with the anti-ApoB100 IgG (1:100) and protein A-Sepharose beads (40 μl wet volume) for 16 h at 4 °C. The protein A-Sepharose beads were washed 5 times with PBS, suspended in Laemmli sample buffer containing 150 mM 2-mercapto-ethanol and heated to 95 °C for 5 min. After electrophoresis on a denaturing 10% polyacrylamide gel, the gel was fixed (10% acetic acid, 30% methanol), treated for fluorography with EN^3^HANCE solution, dried, and X-ray films were exposed at −80 °C for 24 h to 8 days and quantifications were performed with ImageJ analysis program.

### 2.5 Preparation of membrane protein extracts and determination of protein concentrations

Triton X-100 extracts from various tissues prepared from chicken embryos of different developmental stages, from hatched chicks, or from laying hens were prepared as described previously [29]. These samples were used immediately or were quick-frozen in liquid nitrogen and stored at −80 °C until use. Protein concentrations were determined by the method of Bradford (BioRad, Hercules, California, USA) with bovine serum albumin as standard.

### 2.6 SDS-polyacrylamide gel electrophoresis and immunoblotting

For Western blotting, aliquots of extracts were subjected to 10% SDS-PAGE under reducing or non-reducing conditions, and the separated proteins were electrophoretically transferred to nitrocellulose membranes (Hybond-C Extra, Amersham Biosciences, Little Chalfont, UK). The amounts loaded were monitored by Ponceau-S staining of the membranes. Nonspecific binding sites were blocked with TBS (20 mM Tris–HCl, pH 7.4, and 137 mM NaCl) containing 5% (w/v) nonfat dry milk and 0.1% Tween-20 (blocking buffer) for 1 h at room temperature. GgMTP was detected with rabbit anti-ggMTP antiserum (1:5000) followed by incubation with HRP-conjugated goat anti-rabbit IgG (1:50,000, Sigma) and development with the enhanced chemiluminescence protocol (Pierce, Rockford, Illinois, USA). Quantifications were performed with AlphaEaseFC analysis program. The sizes of proteins were estimated with a Precision Plus Protein molecular mass standard (10–250 kDa) from Bio-Rad (Hercules, California, USA).

### 2.7 Preparation of total RNA, cDNA synthesis, PCR, DNA cloning, and sequencing

Total RNA from the indicated galline tissues was extracted using the NucleoSpin RNA II kit (Macherey-Nagel, Dueren, Germany) following the manufacturer's instructions. These samples were used immediately or stored at −80 °C until use. Single-stranded cDNA was synthesized using SuperScript II reverse transcriptase (Invitrogen, Carlsbad, California, USA) and oligo(dT)18-primer. PCR amplification was performed with a T3000 Thermocycler (Biometra, Goettingen, Germany) with a touch-down program using the High Fidelity PCR Enzyme Mix (Fermentas, St. Leon-Rot, Germany). The amplified products were subjected to 1% agarose gel electrophoresis and stained with ethidium bromide. Subsequently, the PCR product was excised from the gel, and DNA was purified with Xact Extraction Kit (GenXpress, Wiener Neudorf, Austria). The purified product was cloned into the pCR2.1-TOPO vector with the TOPO TA Cloning kit (Invitrogen, Carlsbad, California, USA), and subsequently transformed into *E. coli* Top10 cells. The plasmids were isolated using FastPlasmid Mini Kit (5Prime, Hilden, Germany). Plasmids were sent to Agowa GmbH, Berlin, Germany for sequence analysis. The following *G. gallus* (gg)MTP-specific primers were used: forward primer 5′-GCT AGC CTT TTC CAG CTA C-3′ and reverse primer 5′-ATT TTG GCA CCT GTT TTT CG-3′.

### 2.8 Quantitative real time PCR

Quantitative Real Time PCR (qPCR) was performed with the LightCycler 480 system (Roche, Manheim, Germany) using the LightCycler FastStart DNA Master SYBR Green I kit (Roche, Manheim, Germany). The following primers were used: ggMTP, forward primer 5′-GCT AGC CTT TTC CAG CTA C-3′ and reverse primer 5′-ATT TTG GCA CCT GTT TTT CG-3′; and ggβ-actin, forward primer 5′-AGC TAT GAA CTC CCT GAT GG-3′ and reverse primer 5′-ATC TCC TTC TGC ATC CTG TC-3′.

Diluted cDNA samples were used for all qPCR reactions. Serial dilutions of 10^−1^–10^−9^ of the target PCR products were freshly prepared. All samples were analyzed in triplicate measurement ±S.E. from three different incubated eggs or animals. The relative quantification in gene expression was determined using 2^−ΔΔCt^ method [30]. Using this method, the -fold changes in gene expression normalized to an internal control gene are obtained. Here, chicken β-Actin mRNA levels were measured as housekeeping gene and used for normalization. Values are expressed in arbitrary units (AU).

### 2.9 Immunocytochemistry

Freshly isolated tissues were used for paraffin- or cryo-sections. For paraffin sections, tissues were fixed overnight in 4% paraformaldehyde and embedded in paraffin using an Excelsior embedding machine. Sections of 8 μm were cut on a LeicaRM2155 microtome, fixed on Polysine slides (Menzel-Glaeser, Braunschweig, Germany), and dried o/n at 37 °C or for 1 h at 50 °C. The slices were deparaffinized in xylol exchange medium XEM-200 (Vogel, Giessen, Germany) by gentle shaking for 20 min. For rehydration, the tissues were washed consecutively in 100%, 90%, 70%, 50%, and 30% ethanol.

Nonspecific binding of antibodies was inhibited by blocking with PBS containing 1% BSA and 3% inactivated goat serum for 1 h at RT. The sections were incubated overnight at 4 °C with the appropriate antibodies in blocking solution (rabbit anti ggMTP 1:100, rabbit pre-immune serum 1:100). The sections were rinsed 3 × 5 min in PBS and incubated with biotinylated goat α-rabbit secondary antibody (1:500, Sigma) for 1 h at room temperature. After rinsing 3 × 5 min in PBS, the slides were incubated with Streptavidin Peroxidase Polymer (1:200, Sigma, Sigma–Aldrich, Vienna, Austria) for 20 min. For the color reaction, the sections were incubated with AEC (3-amino-9-ethylcarbazol) substrate-chromogen (ready-to-use solution, DAKO, Vienna, Austria). The specimens were mounted in Glycergel Mounting Medium (DAKO, Vienna, Austria) and analyzed by light microscopy (Axiovert 10, Zeiss, Jena, Germany).

Cryosections were prepared from frozen tissue, embedded in O.C.T. Compound Tissue-Tek from Sakura at −24 °C. Sections of 7 μm were cut on a HM500OM microtome, fixed on SuperFrost Plus slides (Menzel-Glaeser, Braunschweig, Germany) and stored at −20 °C. For specific detection of proteins, the sections were incubated at room temperature for 10 min, followed by incubation in ice-cold acetone–methanol 1:1 for 5 min with gentle shaking. After rinsing 3 × 5 min in PBS, the slides were incubated with the appropriate primary antibody as described above, at 4 °C in a humid chamber overnight. After rinsing 3 × 5 min in PBS, the slides were incubated with the secondary biotinylated goat-anti-rabbit antibody as described above.

## 3 Results

To gain insight into MTP expression by the avian embryo, we prepared liver, kidney and small intestine samples from different stages of embryonic development (10, 15 and 20 days), and from 1 and 3 day old chicks, respectively. Unfortunately, due to technical reasons, it was not possible to obtain these organs before day 10 of development. In all experiments, tissues from mature laying hens were used as controls.

**Fig. 1:**
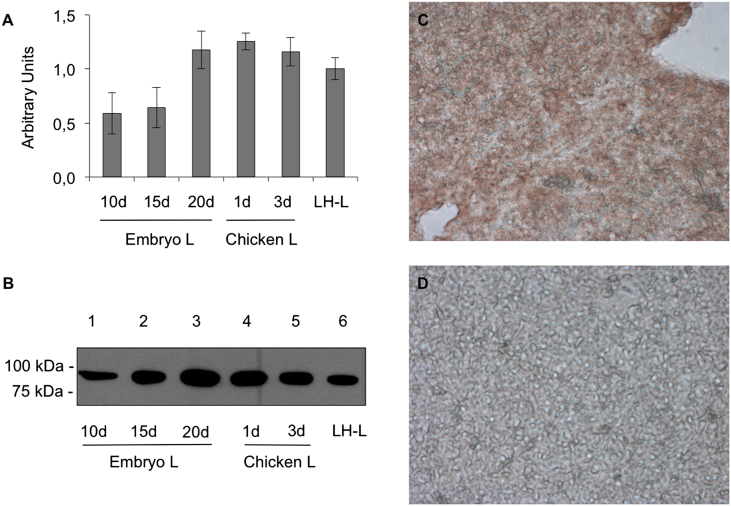
Expression of MTP in the developing chicken liver. A, transcript levels of chicken MTP in the liver were determined using qPCR as described in “Materials and methods section”. Transcript levels are expressed as arbitrary units relative to those of chicken β-actin. B, analysis of chicken MTP protein levels by Western blotting. 10 μg of protein per lane from the liver of the indicated developmental stages were separated by 10% SDS-PAGE under non-reducing conditions, blotted to nitrocellulose membranes and probed with antiserum directed against MTP, followed by an HRP-coupled anti-rabbit secondary antibody as described in “Materials and methods section”. C and D, cryosections of the liver from 17-day-old chicken embryos were prepared as described in “Materials and methods section”. The sections were stained with our rabbit anti-ggMTP antiserum (C) or with corresponding pre-immune serum (D). Images were acquired using a light microscope. Magnification, 40-fold.

First, we analyzed MTP expression in the liver of embryos and hatched chicks (Fig. 1). As determined by qPCR, MTP transcript levels in embryonic liver were detectable at the earliest time point analyzed and rose steadily in the last trimester of embryogenesis (after day 15, see Fig. 1A). One day after hatching, hepatic MTP mRNA was still high, and then appeared to decrease slowly after hatching, as observed in 3-day old chicks to the level observed in mature laying hens. We also performed Western blot analysis of liver membrane protein extracts from animals at the same developmental stages using our rabbit antiserum against chicken MTP (Fig. 1B). The levels of MTP protein in general were in good agreement with the transcript levels, with similar levels before (20 d) and after hatching (1 d), and thereafter approached that in the liver of laying hens. Western blotting results were quantified as described in “Materials and Methods” to determine the MTP protein levels compared to that in laying hen liver. Next, immunohistochemistry of embryonic liver from a 17-day old embryo with the anti-ggMTP antiserum was performed (Fig. 1C), which revealed a high level of MTP in the embryonic liver, in accordance with the results obtained by qPCR and Western blot analysis. No staining was observed when sections were incubated with pre-immune serum (Fig. 1D).

**Fig. 2:**
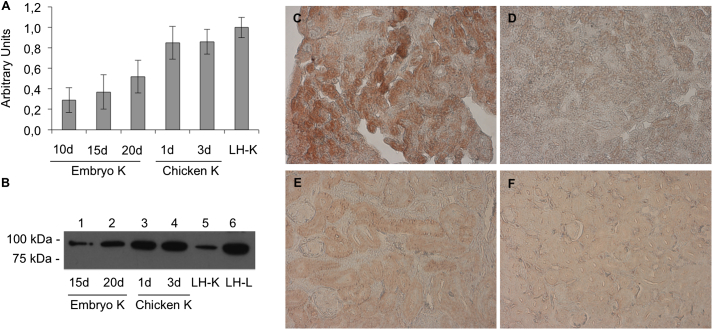
Expression of MTP in the chicken kidney during development. Transcript levels of MTP (A) in the kidney were determined using qPCR as described in “Materials and methods section”. Transcript levels are expressed as arbitrary units relative to those of chicken β-actin. B, analysis of chicken MTP protein levels by Western blotting. 25 μg of protein per lane from the kidneys of the indicated developmental stages were separated by 10% SDS-PAGE under non-reducing conditions, blotted to nitrocellulose membranes and probed with antiserum directed against MTP, followed by an HRP-coupled anti-rabbit secondary antibody as described in “Materials and methods section”. Cryosections of the kidney from 17-day-old chicken embryos (C and D) and from laying hen (E and F) were prepared as described in “Materials and methods section”. The sections were stained with our rabbit anti-ggMTP antiserum (C and E) or with corresponding pre-immune serum (D and F). Images were acquired using a light microscope. Magnification, 40-fold.

To analyze MTP transcript levels in the kidney of embryos and hatched chicks, quantitative PCR was performed (Fig. 2A). The levels of mRNA remained constant from day 10–20 of embryogenesis, and increased after hatching. Next, the membrane protein extracts from embryonic kidneys from 15- and 20-day old embryos and from 1- and 3-day old chicken were subjected to Western blot analysis using our anti-ggMTP antiserum (Fig. 2B). In accordance to the quantitative PCR results, the MTP protein levels increased after hatching. In the particular laying hen analyzed for protein levels, expression of MTP was higher in liver than in kidney. The immunohistochemical staining of the kidney of a 17-day old embryo and of a laying hen with anti-ggMTP antiserum (Fig. 2C and E) demonstrate the expression of MTP in the embryonic kidney. When sections were incubated with pre-immune serum (Fig. 2D and F), no staining was observed.

**Fig. 3:**
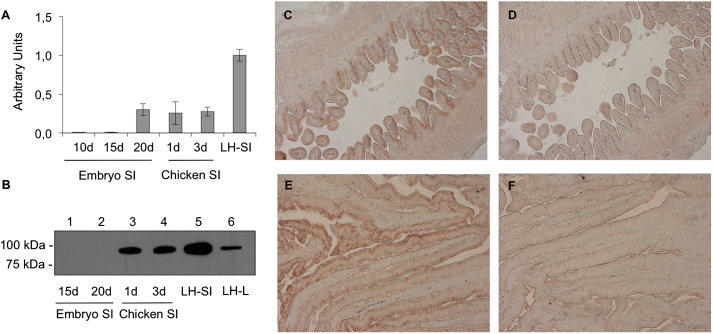
Expression of MTP in the small intestine of the developing chicken. A, transcript levels of MTP in the small intestine were determined using qPCR as described in “Materials and methods section”. Transcript levels are expressed as arbitrary units relative to those of chicken β-actin. B, analysis of MTP protein in the small intestine by Western blotting. 10 μg per lane of protein from livers of the indicated developmental stages were separated by 10% SDS-PAGE under non-reducing conditions, blotted to nitrocellulose membranes and probed with antiserum directed against MTP, followed by an HRP-coupled anti-rabbit secondary antibody as described in “Materials and methods section”. Cryosections of the small intestine parallel to the villi from 17-day-old chicken embryos (C and D) and from laying hen (E and F) were prepared as described in “Materials and methods section. The sections were stained with our rabbit anti-ggMTP antiserum (C and E) or with corresponding pre-immune serum (D and F). Images were acquired using a light microscope. Magnification, 40-fold.

The quantitative analysis of MTP mRNA in small intestine of embryos and hatched chicks showed very low transcript levels on embryonic days 10 and 15, followed by a dramatic increase towards day 20; the level remained stable for at least 3 days after hatching (Fig. 3A). In mature hens, expression levels rose an additional 3-fold. Western blot analysis showed that MTP protein levels in embryonic small intestine likely rise only shortly before hatching and are then clearly detectable after hatching (days 1 and 3). In accordance with the results obtained by qPCR, MTP protein was substantially elevated in laying hens (Fig. 3B). The immunohistochemical staining of embryonic intestine from a 17 days old embryo (Fig. 3C) and a laying hen (Fig. 3E) with anti-ggMTP antiserum revealed the presence of MTP in the enterocytes lining the villi. No staining was observed when the sections were incubated with pre-immune serum (Fig. 3D and F).

**Fig. 4:**
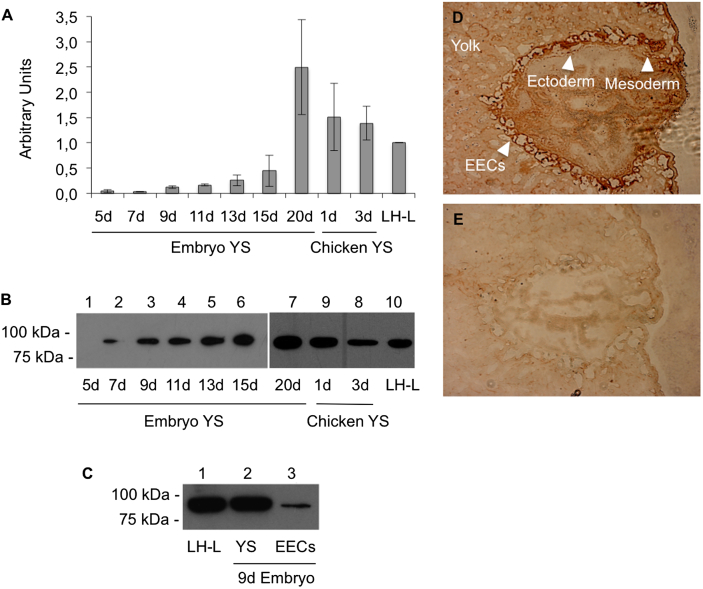
Expression of MTP in the developing chicken yolk sac. A, transcript levels of MTP in the chicken YS at the indicated day of development (*d*) and 1 and 3 days after hatching were determined using qPCR as described in “Materials and methods section”. Transcript levels are expressed as arbitrary units relative to those of chicken β-actin. B and C, analysis of MTP protein levels by Western blotting. 10 μg (B) or 50 μg (C) of protein per lane from the YS obtained at the indicated developmental stages and the indicated tissues (LH-L, laying hen liver; EECs, endodermal epithelial cells) were separated by 10% SDS-PAGE under non-reducing conditions, blotted to nitrocellulose membranes and probed with our rabbit antiserum directed against chicken MTP, followed by an HRP-coupled anti-rabbit secondary antibody as described in “Materials and methods section”. D and E, cryosections of the YS tissue from 15-day-old chicken embryos were prepared as described in “Materials and methods section”. The sections were stained with our rabbit anti-ggMTP antiserum (D) or with corresponding pre-immune serum (E). The position of the three germ layers are indicated by arrowheads. Images were acquired using a light microscope. Magnification, 40-fold.

We also determined the transcript levels of chicken MTP in the YS during days 5–20 of embryo development, and from 1- and 3-day old chicks, respectively. As positive control laying hen liver was used. As shown in Fig. 4A, MTP RNA expression starts at the end of the first trimester of embryonic development, i.e., on or about day 7 of incubation, increases gradually during the growth of the embryo, and is observed in the YS for at least 3 days after hatching. The highest expression was found in the YS of 20-day old embryos, i.e., at the end of embryonic development. Anti-ggMTP antiserum was used to analyze the MTP protein levels by Western blotting of YS extracts from the stages also analyzed at the transcript levels (Fig. 4B). As observed for mRNA, the levels of YS MTP protein increased during embryo development, and MTP was still present in the YS after hatching. The levels of MTP protein in the YS are in accordance with the results obtained by qPCR. The YS consists of three germ layers, yolk sac ectoderm, mesoderm, and endoderm [31]. Immunohistochemical staining of YS sections of 15-day old embryos with anti-ggMTP antiserum revealed that MTP is predominantly localized in the inner endodermal epithelial layer of YS facing the yolk (Fig. 4D), as also shown by Western blot analysis (Fig. 4C) and in our previous work [26]. No specific immunostaining of sections was observed they were incubated with pre-immune serum (Fig. 4E).

**Fig. 5:**
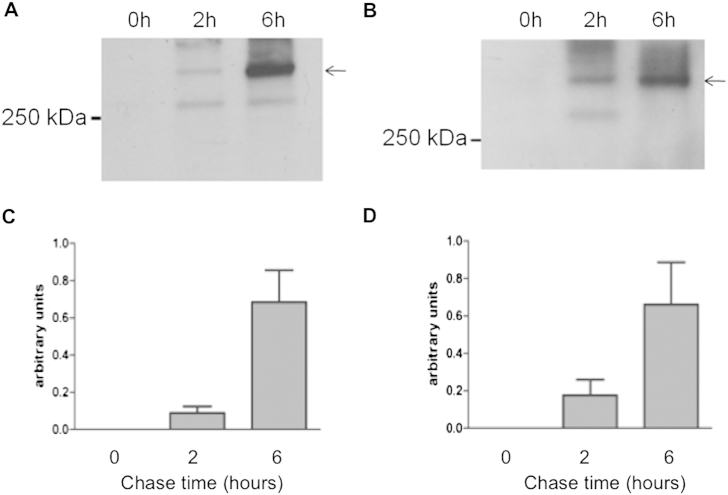
ApoB secretion by the chicken yolk sac. YS tissue explants from 15-day old (A, C) and 20-day old chicken embryos (B, D) were pulse-labeled with [^35^S]methionine/cysteine for 1 h and chased for the indicated periods. Radiolabeled secreted apoB was immunoprecipitated with our rabbit antiserum directed against chicken apoB from the conditioned medium, and the results were quantified (C, D) as described in “Materials and methods section”.

Finally, to test for *de novo* synthesis of apoB by the YS, pulse/chase analysis with cultured YS fragments of 15-day (Fig. 5A) and 20-day (Fig. 5B) old embryos was performed. Newly synthesized radiolabeled apoB100 was immunoprecipitated from the medium and subjected to SDS-PAGE and autoradiographic analysis. A strong signal corresponding to apoB-100 is detectable after a 6-hr chase period in cultured YSs from both 15- (Fig. 5C) and 20-day old (Fig. 5D) embryos.

## 4 Discussion

Our findings support the notion that apoB100 is synthesized *de novo* and secreted by the chicken yolk sac. Therefore, co-expression of MTP indicates that it is also important for these processes in the yolk sac. Previous studies have shown that apoB100 of plasma VLDL in the laying hen undergoes partial proteolytic cleavage upon receptor-mediated endocytosis into the oocyte, where it is stored as yolk VLDL [32,33]. The characteristics of apoB in plasma VLDL particles of adult animals and embryos are different from those of VLDL particles found in yolk [27]. Yolk VLDL contains immunoreactive proteolytic fragments of apoB [32,33], whereas intact apoB100 is present in the circulating plasma VLDL in embryos and adult animals [27,32]. The YS has been suggested to be the major source of lipoproteins and the site of cholesterol esterification [34–36]. Previous studies have shown the presence of apoB mRNA in YS [37], and biochemical evidence has suggested that VLDL particles are synthesized in YS [26,27].

The demonstration that MTP is produced during embryonic development in the YS as well as in other organs known to secrete lipoproteins supports the hypothesis that lipoproteins synthesized in the embryo also contribute to the embryonic plasma pool of apoB-containing lipoproteins. These tissues are the liver, small intestine, and, in contrast to mammals, the kidney. Thus, we analyzed these four organs during different developmental stages for MTP expression. There is ample evidence for the necessity of co-expression of MTP and ApoB for the assembly of apoB into triglyceride-rich lipoprotein particles [3–5]; for review see Ref. [38]. VLDL assembly and secretion by the liver have been extensively analyzed in the chicken, since the biochemical and physiological properties of the circulating particles are dramatically influenced by estrogens [37,39–46]. Lazier et al. showed apoB mRNA expression in the embryonic liver from day 6.5 of development [37]. Tarugi at al. observed a large hepatic cholesteryl ester store in the third trimester of embryonic development, which intermittently increased at hatching and decreased within a few days thereafter [47,48]. Noble at al. suggested that the accumulation of cholesteryl esters in the liver of late embryo derives from the uptake of cholesterol(ester)-rich lipoproteins secreted from YS [36,49]. Our results complement these observations, as increased MTP and apoB production in embryonic liver is compatible with the utilization of the accumulated cholesterol for hepatic *de-novo* synthesis of lipoproteins.

The complex process of absorption of dietary lipids by the enterocytes and their subsequent secretion as components of lipoproteins into the circulatory system is also dependent on apoB and MTP (for review see Ref. [19]). In mammals, the major lipoproteins secreted by the intestine are chylomicrons, which transport dietary fat and fat-soluble vitamins into the blood for distribution to target tissues. A key structural component of mammalian chylomicrons is apoB48. Since chickens do not possess the machinery required for the production of apoB48, so-called portomicrons, the triglyceride-rich lipoproteins considered to be equivalent to chylomicrons [14,50], harbor apoB100. Different from the embryonic liver, we determined that the MTP transcript levels in small intestine rose from negligible to substantial only shortly before hatching, and that MTP protein is detectable in the intestine only after hatching (Fig. 3). These findings extend the work by Lazier et al. who reported a lack of apoB mRNA in the embryonic intestine before hatching [37]. This implies that in contrast to the liver with its considerable pool of lipids accumulating during embryogenesis, the embryonic intestinal cells lacking this large pool are capable of lipoprotein synthesis and secretion only just before and/or after hatching.

Besides the liver and small intestine, the kidney is a major lipoprotein-producing organ in the chicken [46,51], which unlike mammals, synthesize renal apoB at significant levels [40,52]. Lazier et al. [37] have shown apoB mRNA in the embryonic kidney starting at day 7.5 of development. Our data demonstrate increasing levels of MTP mRNA and protein in the kidney during embryogenesis, with a further increase after hatching. Thus, the embryonic kidney, like the liver, but in contrast to the intestine, appears to have the capacity to serve as source of lipoproteins in the embryo. Immunohistochemistry of embryonic liver, small intestine, and kidney confirmed the presence of MTP in the respective tissues, and also provided insights into the localization of MTP in these tissues.

Taken together, our present findings strengthen the notion that the synthesis and secretion of lipoproteins from embryonic tissues is not limited to the yolk sac, but also is a physiologically important feature of the developing liver, kidney, and, to a limited extent, the intestine.
